# miRNA-558 promotes gastric cancer progression through attenuating Smad4-mediated repression of heparanase expression

**DOI:** 10.1038/cddis.2016.293

**Published:** 2016-09-29

**Authors:** Liduan Zheng, Wanju Jiao, Huajie Song, Hongxia Qu, Dan Li, Hong Mei, Yajun Chen, Feng Yang, Huanhuan Li, Kai Huang, Qiangsong Tong

**Affiliations:** 1 Department of Pathology, Union Hospital, Tongji Medical College, Huazhong University of Science and Technology, 1277 Jiefang Avenue, Wuhan 430022, Hubei Province, P. R. China; 2 Clinical Center of Human Genomic Research, Union Hospital, Tongji Medical College, Huazhong University of Science and Technology, 1277 Jiefang Avenue, Wuhan 430022, Hubei Province, P. R. China; 3 Department of Surgery, Union Hospital, Tongji Medical College, Huazhong University of Science and Technology, 1277 Jiefang Avenue, Wuhan 430022, Hubei Province, P. R. China

## Abstract

Previous studies have indicated that as the only mammalian endo-*β*-D-glucuronidase, heparanase (HPSE) is up-regulated and associated with poor prognosis in gastric cancer, while the underlying mechanisms still remain to be determined. Herein, through integrative analysis of public datasets, we found microRNA-558 (miR-558) and SMAD family member 4 (Smad4) as the crucial transcription regulators of HPSE expression in gastric cancer, with their adjacent target sites within the promoter of *HPSE*. We identified that endogenous miR-558 activated the transcription and expression of HPSE in gastric cancer cell lines. In contrast, Smad4 suppressed the nascent transcription and expression of HPSE via directly binding to its promoter. Mechanistically, miR-558 recognized its complementary site within *HPSE* promoter to decrease the binding of Smad4 in an Argonaute 1-dependent manner. Ectopic expression or knockdown experiments indicated that miR-558 promoted the *in vitro* and *in vivo* tumorigenesis and aggressiveness of gastric cancer cell lines via attenuating Smad4-mediated repression of HPSE expression. In clinical gastric cancer specimens, up-regulation of miR-558 and down-regulation of Smad4 were positively correlated with HPSE expression. Kaplan–Meier survival analysis revealed that miR-558 and Smad4 were associated with unfavourable and favourable outcome of gastric cancer patients, respectively. Therefore, these findings demonstrate that miR-558 facilitates the progression of gastric cancer through directly targeting the *HPSE* promoter to attenuate Smad4-mediated repression of HPSE expression.

As the fifth most common malignancy, gastric cancer is currently one of the leading causes of death around the world.^1^ In spite of achievement in surgery and multimodal therapy, the outcome of gastric cancer in advanced stages is still dismal mainly due to tumour growth and progression.^1^ Therefore, it is an urgent duty to elucidate the mechanisms underlying the tumorigenesis and aggressiveness of gastric cancer.^2^ Heparanase (HPSE), the only mammalian endo-*β*-D-glucuronidase, plays crucial roles in the degradation of extracellular components and release of angiogenic and growth-promoting factors, thus facilitating tumour growth, invasion, metastasis and angiogenesis.^3, 4^ In addition, HPSE promotes the expression of vascular endothelial growth factor (VEGF) through activation of Src pathway.^5^ In most of human cancers, HPSE is up-regulated and associated with tumour aggressiveness and unfavourable outcome.^6, 7^ High expression of HPSE has been documented in gastric cancer specimens, which is associated with poor outcome of patients,^8^ indicating the essential functions of *HPSE* in the tumorigenesis and aggressiveness of gastric cancer.

The expression of human *HPSE* gene is regulated by transcription factors.^9^ For example, v-ets avian erythroblastosis virus E26 oncogene homolog and specific protein 1 are essential for the basal *HPSE* transcription, whereas early growth response gene 1 contributes to the inducible transcription of *HPSE* in human cancer cell lines and T lymphocytes.^10, 11, 12, 13^ In addition, cAMP responsive element binding protein regulates the expression of HPSE in brain-metastatic melanoma cells.^14^ In breast carcinomas, estrogen receptor contributes to estrogen-induced transcriptional activation of *HPSE*.^15^ On the other hand, tumour suppressor p53 is able to bind to the promoter of *HPSE* to inhibit its expression in cancer cells.^16^ However, the transcriptional regulators and underlying mechanisms essential for HPSE expression in gastric cancer remain to be elucidated.

In this study, through integrative analysis of the *cis*-regulatory elements and public datasets of chromatin immunoprecipitation (ChIP) and microarray, we identified microRNA-558 (miR-558) and SMAD family member 4 (Smad4) as crucial transcriptional regulators of HPSE expression in gastric cancer, with their adjacent target sites within the *HPSE* promoter. For the first time to our knowledge, we demonstrate that miR-558 facilitates the HPSE expression via transcriptional activation in gastric cancer cell lines. In contrast, Smad4 represses the transcription of *HPSE* through directly binding to its promoter. Mechanistically, miR-558 recognizes its complementary site within *HPSE* promoter to decrease the binding of Smad4 in an Argonaute 1 (AGO1)-dependent manner, thus facilitating the *in vitro* and *in vivo* tumorigenesis and progression of gastric cancer cells, indicating the oncogenic functions of miR-558 in gastric cancer.

## Results

### miR-558 facilitates the HPSE expression in gastric cancer cells

**Figure 1:**
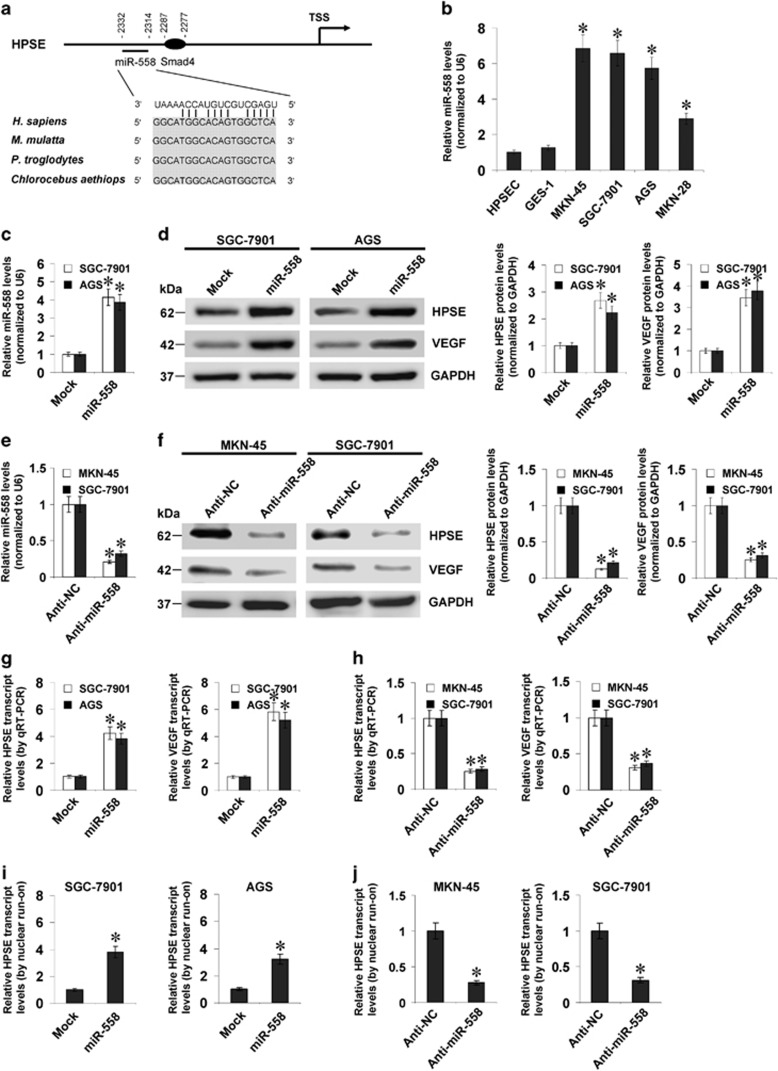
miR-558 facilitates the HPSE expression in gastric cancer cells. (**a**) Scheme of potential target sites of Smad4 (−2277/−2287) and miR-558 (−2314/−2332) within the promoter of *HPSE*. (**b**) Real-time quantitative RT-PCR assay showing the miR-558 levels in primary stomach epithelial HPSEC cells, SV40-immortalized normal gastric epithelial GES-1 cells and gastric cancer cell lines (MKN-45, SGC-7901, AGS and MKN-28). (**c**) and (**d**) Real-time quantitative RT-PCR and western blot assays indicating the expression of miR-558, HPSE and VEGF in gastric cancer cells stably transfected with empty vector (mock) or miR-558 precursor. (**e**) and (**f**) Real-time quantitative RT-PCR and western blot assays showing the expression of miR-558, HPSE and VEGF in gastric cancer cells transfected with negative control inhibitor (anti-NC, 100 nmol/l) or anti-miR-558 inhibitor (100 nmol/l). (**g**) and (**h**) Real-time quantitative RT-PCR assay indicating the transcript levels of *HPSE* and *VEGF* in gastric cancer cells transfected with mock, miR-558 precursor, anti-NC (100 nmol/l) or anti-miR-558 inhibitor (100 nmol/l). (**i**) and (**j**) Nuclear run-on assay showing the nascent *HPSE* transcript levels in gastric cancer cells transfected with mock, miR-558 precursor, anti-NC (100 nmol/l) or anti-miR-558 inhibitor (100 nmol/l). *P<0.01 versus HPSEC, mock or anti-NC

To investigate the regulators crucial for the expression of HPSE in gastric cancer, we analysed the potential binding sites of transcription factor within its promoter, using computational algorithm programmes. Over-lapping analysis of Genomatrix^17^ and PROMO^18^ revealed the potential binding site of Smad4 (-2287/-2277 upstream the transcription start site) within *HPSE* promoter region (chr4:84255936-84259422; Figure 1a and Supplementary Figure S1A). Further analysis of chromatin immunoprecipitation sequencing (ChIP-seq) dataset (GSE27526)^19^ revealed the enrichment of Smad4 within this region (Supplementary Figure S1A). In addition, analysis of microPIR database^20^ implicated that miR-558 targeting site with high complementarity was noted at −2332/−2314 bp region surrounding that of Smad4 (Figure 1a). Notably, mining the publicly available Gene Expression Omnibus (GEO) datasets indicated the negative correlation between Smad4 and HPSE levels in different gastric cancer cohorts (Supplementary Figure S1B). Moreover, as the host gene of miR-558,^21^ baculoviral IAP repeat containing 6 (BIRC6) was positively correlated with HPSE levels in gastric cancer cases derived from Gene Expression Omnibus datasets (Supplementary Figure S1C). Elevated miR-558 levels were detected in gastric cancer cells, when comparing with those of primary stomach epithelial cells (Figure 1b).

To address the regulatory roles of miR-558 in *HPSE* transcription, we observed the impacts of miR-558 on HPSE levels in cultured gastric cancer cell lines with different miR-558 levels. We applied the SGC-7901 and AGS cells for over-expression experiments, and chose the MKN-45 and SGC-7901 cells for knockdown studies. The miR-558 precursor was stably transfected into gastric cancer cells, resulting in increase of miR-558 and HPSE levels, than those in empty vector (mock)-transfected cells (Figures 1c and d). Meanwhile, transfection of anti-miR-558 inhibitor obviously decreased the expression of miR-558 and HPSE in MKN-45 and SGC-7901 cells, than those treated by negative control inhibitor (anti-NC; Figures 1e and f). Moreover, ectopic expression or knockdown of miR-558 led to increase and decrease in the nascent transcription and transcript levels of *HPSE* in gastric cancer cell lines, respectively (Figures 1g–j). The expression of *VEGF*, the HPSE downstream gene,^5^ was obviously increased or decreased in miR-558 over-expressing and knockdown gastric cancer cells (Figures 1d and f–h). However, no miR-558 targeting site was noted within the *VEGF* promoter by analysing the microPIR database.^20^ Ectopic expression or knockdown of miR-558 did not influence the promoter activity of *VEGF* in gastric cancer cells (Supplementary Figure S2A), indicating no direct regulation of *VEGF* transcription by miR-558. These data indicated that miR-558 increased the expression of HPSE in gastric cancer cells.

### miR-558 activates the promoter activity and transcription of *HPSE* in an AGO1-dependent manner

**Figure 2:**
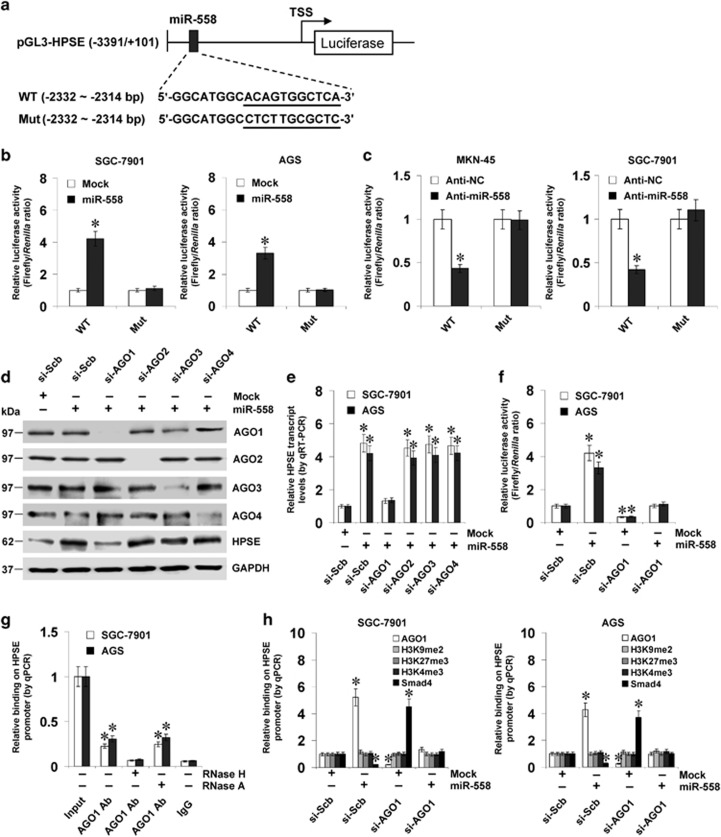
miR-558 activates the promoter activity and transcription of *HPSE* in an AGO1-dependent manner. (**a**) Scheme and sequence of the wild type (WT) and mutant (Mut) miR-558 binding site within the luciferase reporter of *HPSE* promoter. (**b**) and (**c**) Dual-luciferase assay showing the activity of *HPSE* promoter and its mutant in SGC-7901 and AGS cells transfected with empty vector (mock), miR-558 precursor, negative control inhibitor (anti-NC, 100 nmol/l) or anti-miR-558 inhibitor (100 nmol/l). (**d**) and (**e**) Western blot and real-time quantitative RT-PCR assays indicating the expression of AGO1, AGO2, AGO3, AGO4 and HPSE in gastric cancer cells transfected with mock or miR-558 precursor, and those co-transfected with scramble siRNA (si-Scb) or siRNAs specific for *AGO1*, *AGO2*, *AGO3* or *AGO4*. (**f**) Dual-luciferase assay showing the *HPSE* promoter activity in gastric cancer cells stably transfected with mock or miR-558 precursor, and those co-transfected with si-Scb or si-AGO1. (**g**) ChIP and qPCR assay indicating the binding of AGO1 to *HPSE* promoter in gastric cancer cells treated with RNase H or RNase A. (**h**) ChIP and qPCR assay showing the enrichment of AGO1, H3K9me2, H3K27me3, H3K4me3 and Smad4 on *HPSE* promoter in SGC-7901 and AGS cells transfected with mock or miR-558 precursor, and those co-transfected with si-Scb or si-AGO1. *P<0.01 versus mock, anti-NC, mock+si-Scb or IgG

To investigate whether miR-558 could target the *HPSE* promoter to increase its transcription, gastric cancer cells were transfected with the luciferase reporter of *HPSE* promoter and its mutant (Figure 2a). Over-expression or knockdown of miR-558 enhanced and attenuated the activity of *HPSE* promoter, respectively (Figures 2b and c), which was attenuated by mutation of miR-558 targeting site (Figures 2b and c). Since AGO1 is involved in miR-558-activated transcription of *HPSE*,^22^ small interfering RNAs (siRNAs) specific for AGOs were introduced into SGC-7901 and AGS cells. Knockdown of *AGO1*, but not of Argonaute 2 (*AGO2*), Argonaute 3 (*AGO3*) or Argonaute 4 (*AGO4*), abolished the miR-558-facilitated protein and transcriptional levels of *HPSE* in gastric cancer cells (Figures 2d and e). In addition, knockdown of *AGO1* attenuated the increase in promoter activity and nascent transcription of *HPSE* induced by over-expression of miR-558 in gastric cancer cells (Figure 2f and Supplementary Figure S2B). The ChIP and real-time quantitative PCR (qPCR) assay revealed that in cultured gastric cancer cells, enrichment of AGO1 was observed at the region (−2347/−2148 bp), which was surrounding the binding site of miR-558 (Figure 2g). In addition, treatment of gastric cancer cells with RNase H, but not with RNase A, inhibited the enrichment of AGO1 on the *HPSE* promoter (Figure 2g). Stable over-expression of miR-558 in gastric cancer cells led to enhanced binding of AGO1 and decreased enrichment of Smad4, which was inhibited by knockdown of *AGO1* (Figure 2h), without changes in the enrichment of epigenetic markers histone H3 lysine 9 dimethylation (H3K9me2), histone H3 lysine 27 trimethylation (H3K27me3) or histone H3 lysine 4 trimethylation (H3K4me3) on *HPSE* promoter (Figure 2h). Collectively, these data suggested that miR-558 recognized the target site to activate the *HPSE* transcription in an AGO1-dependent manner in gastric cancer cells.

### miR-558 attenuates the Smad4-mediated repression of *HPSE* transcription in gastric cancer cells

**Figure 3:**
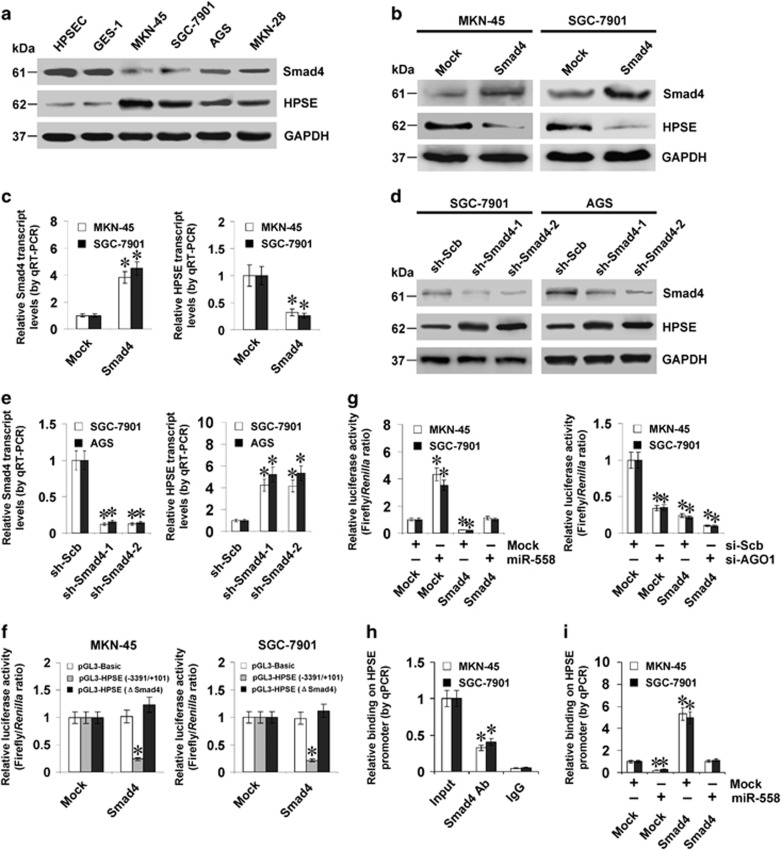
miR-558 attenuates the Smad4-mediated repression of *HPSE* transcription in gastric cancer cells. (**a**) Western blot showing the expression of Smad4 and HPSE in primary stomach epithelial HPSEC cells, SV40-immortalized normal gastric epithelial GES-1 cells and gastric cancer cell lines (MKN-45, SGC-7901, AGS and MKN-28). (**b**) and (**c**) Western blot and real-time quantitative RT-PCR assays indicating the protein and transcript levels of Smad4 and HPSE in MKN-45 and SGC-7901 cells stably transfected with empty (mock) or *Smad4*. (**d**) and (**e**) Western blot and real-time quantitative RT-PCR assays showing the protein and transcript levels of Smad4 and HPSE in SGC-7901 and AGS cells stably transfected with scramble shRNA (sh-Scb) or shRNA specific for *Smad4* (sh-Smad4). (**f**) and (**g**) Dual-luciferase assay indicating the *HPSE* promoter activity in MKN-45 and SGC-7901 cells stably transfected with empty vector (mock) or *Smad4*, and those co-transfected with miR-558 precursor or siRNA specific for *AGO1* (si-AGO1). (**h**) and (**i**) ChIP and qPCR assay showing the binding of Smad4 to the *HPSE* promoter in gastric cancer cells, and those stably transfected with empty vector (mock), Smad4 or miR-558 precursor. *P<0.01 versus mock, sh-Scb, mock+ si-Scb or IgG

To determine the mechanisms essential for miR-558-induced transcriptional activation, we addressed the roles of Smad4 in the expression of HPSE. Lower Smad4 and higher HPSE levels were noted in gastric cancer cell lines, when comparing with primary stomach epithelial cells (Figure 3a and Supplementary Figure S3A). Stable transfection of *Smad4* into MKN-45 and SGC-7901 cells led to increased Smad4 expression and decreased HPSE levels (Figures 3b and c, and Supplementary Figure S3B). On the other hand, stable transfection of short hairpin RNA (shRNA) targeting *Smad4* (sh-Smad4) into SGC-7901 and AGS cells led to reduced Smad4 expression and enhanced HPSE levels (Figures 3d and e, and Supplementary Figure S3C). Ectopic expression of Smad4 decreased the *HPSE* promoter activity in gastric cancer cells (Figure 3f), and mutation of Smad4 binding site abolished these effects (Figure 3f). In addition, transfection of miR-558 precursor or AGO1-specific siRNA (si-AGO1) prevented and facilitated the repression of *HPSE* promoter activity induced by Smad4, respectively (Figure 3g). Moreover, ChIP and real-time qPCR revealed the enrichment of Smad4 around its binding site in gastric cancer cells (Figure 3h). Stable over-expression of miR-558 attenuated the enrichment of Smad4 on *HPSE* promoter induced by ectopic expression of Smad4 in gastric cancer cells (Figure 3i). These data suggested that miR-558 attenuated the Smad4-mediated repression of HPSE levels in gastric cancer cells.

### miR-558 promotes the tumorigenesis and aggressiveness of gastric cancer cells via attenuating Smad4-mediated repression of HPSE expression *in vitro*

**Figure 4:**
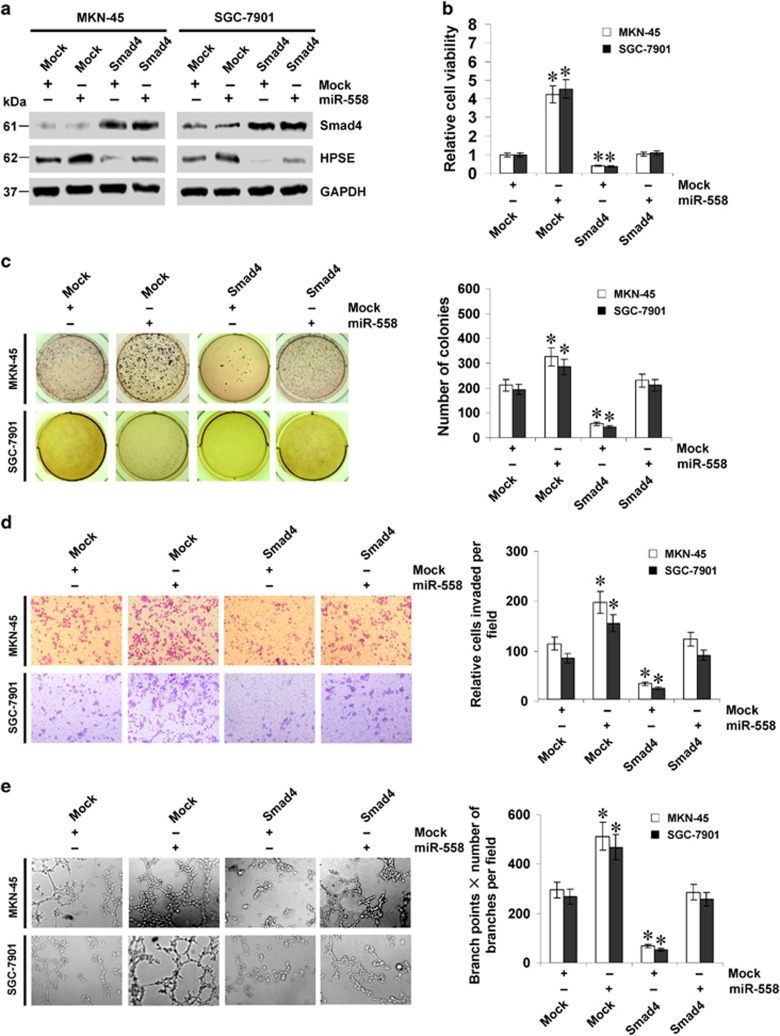
miR-558 promotes the tumorigenesis and aggressiveness of gastric cancer cells through attenuating Smad4-mediated repression of HPSE expression *in vitro*. (**a**) Western blot assay showing the expression of Smad4 and HPSE in MKN-45 and SGC-7901 cells stably transfected with empty vector (mock) or miR-558 precursor, and those co-transfected with *Smad4*. (**b**) MTT colorimetric assay indicating the viability of gastric cancer cells stably transfected with mock or miR-558 precursor, and those co-transfected with *Smad4*. (**c**) Representation (left) and quantification (right) of soft agar assay showing the anchorage-independent growth of MKN-45 and SGC-7901 cells stably transfected with mock or miR-558 precursor, and those co-transfected with *Smad4*. (**d**) Representation (left) and quantification (right) of matrigel invasion assay indicating the invasion capability of gastric cancer cells stably transfected with mock or miR-558 precursor, and those co-transfected with *Smad4*. (**e**) Representation (left) and quantification (right) of tube formation assay showing the angiogenic capability of gastric cancer cells stably transfected with mock or miR-558 precursor, and those co-transfected with *Smad4*. *P<0.01 versus mock

Since the above evidence indicated that miR-558 attenuated the binding of Smad4 to *HPSE* promoter, we further explored the impacts of miR-558 on Smad4-mediated repression of HPSE expression in gastric cancer cells. Ectopic expression of miR-558 restored the decreased HPSE protein levels induced by stable transfection of *Smad4* (Figure 4a). As shown in Figures 4b and c, Smad4 over-expression decreased the viability and growth of MKN-45 and SGC-7901 cells. In matrigel invasion assay, stable over-expression of Smad4 inhibited the invasion capacity of gastric cancer cells (Figure 4d). Treatment of endothelial cells with the medium preconditioned by Smad4 over-expressing gastric cancer cells reduced their tube formation capability (Figure 4e). In addition, ectopic expression of miR-558 rescued the MKN-45 and SGC-7901 cells from decreased viability, growth, invasion and angiogenesis capability induced by stable transfection of *Smad4* (Figures 4b–e). In contrast, stable knockdown of *Smad4* decreased the Smad4 binding to *HPSE* promoter in SGC-7901 and AGS cells (Supplementary Figure S3D), resulting in enhanced HPSE expression (Supplementary Figures S3E and S4A) and increased capability in cell viability (Supplementary Figure S4B), growth (Supplementary Figure S4C), invasion (Supplementary Figure S4D) and angiogenesis (Supplementary Figure S4E). Moreover, down-regulation of miR-558 rescued the gastric cancer cells from *Smad4* knockdown-altered biological features (Supplementary Figures S4B, S4C, S4D and S4E). Meanwhile, knockdown or over-expression of *HPSE* prevented the gastric cancer cells from alteration in the growth, invasion and angiogenesis induced by ectopic expression of miR-558 or *Smad4*, respectively (Supplementary Figure S5). These findings suggested that miR-558 remarkably increased the tumorigenesis and aggressiveness of gastric cancer cells through attenuating Smad4-mediated repression of HPSE expression *in vitro*.

### miR-558 facilitates the tumorigenesis and aggressiveness of gastric cancer cells *in vivo*

**Figure 5:**
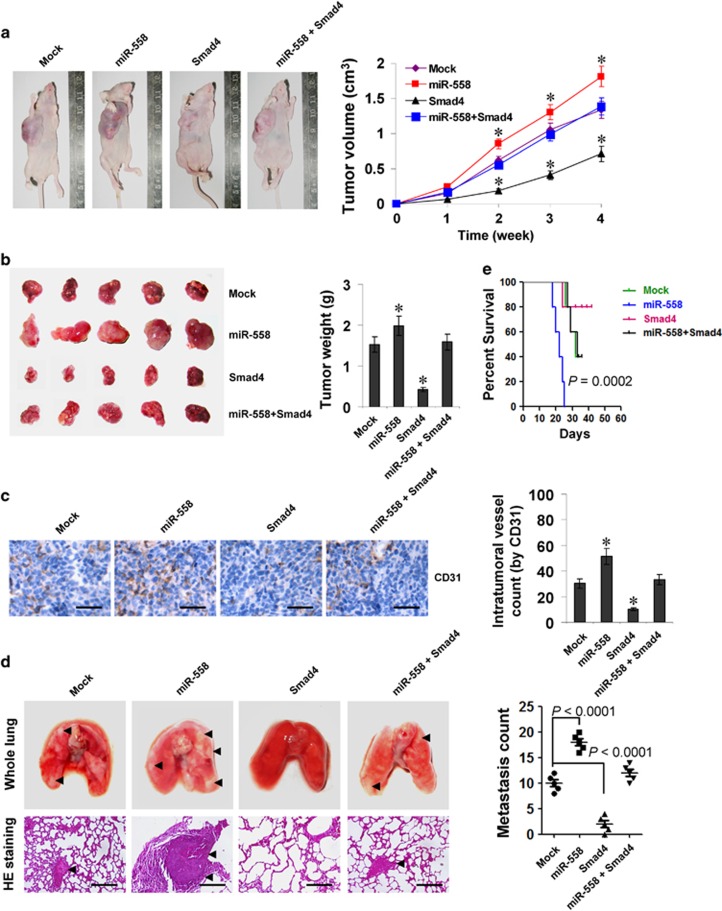
miR-558 facilitates the tumorigenesis and aggressiveness of gastric cancer cells *in vivo*. (**a**) The *in vivo* growth curve of SGC-7901 cells (1 × 10^6^) stably transfected with empty vector (mock) or miR-558 precursor, and those co-transfected with *Smad4* in athymic nude mice (*n*=5 for each group), and xenograft tumours after hypodermic injection for 4 weeks. (**b**) Representation (left) and quantification (right) of xenograft tumours formed by hypodermic injection of SGC-7901 cells stably transfected with mock or miR-558 precursor, and those co-transfected with *Smad4*. (**c**) Immunohistochemical staining (left) and quantification (right) of CD31 expression within tumours formed by hypodermic injection of SGC-7901 cells stably transfected with mock or miR-558 precursor, and those co-transfected with *Smad4*. Scale bars: 100 *μ*m. (**d**) Representation (left, arrowhead) and quantification (right) of lung metastasis of nude mice with injection of SGC-7901 cells (0.4 × 10^6^) stably transfected with mock or miR-558 precursor, and those co-transfected with *Smad4* via the tail vein (*n*=5 for each group). Scale bars: 100 *μ*m. (**e**) Kaplan–Meier survival plots of nude mice with injection of SGC-7901 cells (0.4 × 10^6^) stably transfected with mock or miR-558 precursor, and those co-transfected with *Smad4* into the tail vein of athymic nude mice (*n*=5 for each group). *P<0.01 versus mock

We further explored the impacts of miR-558 on Smad4-mediated repression of tumorigenesis and aggressiveness *in vivo*. Stable over-expression of miR-558 led to increased *in vivo* growth of SGC-7901 cells in athymic nude mice and enhanced weight of subcutaneous xenograft tumours (Figures 5a and b). Meanwhile, the intratumoral CD31-positive microvessels and mean vessel density were also increased (Figure 5c). In the experimental metastasis studies, SGC-7901 cells stably transfected with miR-558 precursor established significantly more lung metastatic colonies and lower survival probability in athymic nude mice, than those transfected with empty vector (mock) (Figures 5d and e). Moreover, stable over-expression of miR-558 in SGC-7901 cells rescued the Smad4-inhibited growth, metastasis, angiogenesis and survival duration in athymic nude mice (Figures 5a–e). These data indicated that miR-558 could facilitate the tumorigenesis and aggressiveness of gastric cancer cells *in vivo*.

### Smad4 and miR-558 are inversely or positively correlated with HPSE expression in gastric cancer tissues

**Figure 6:**
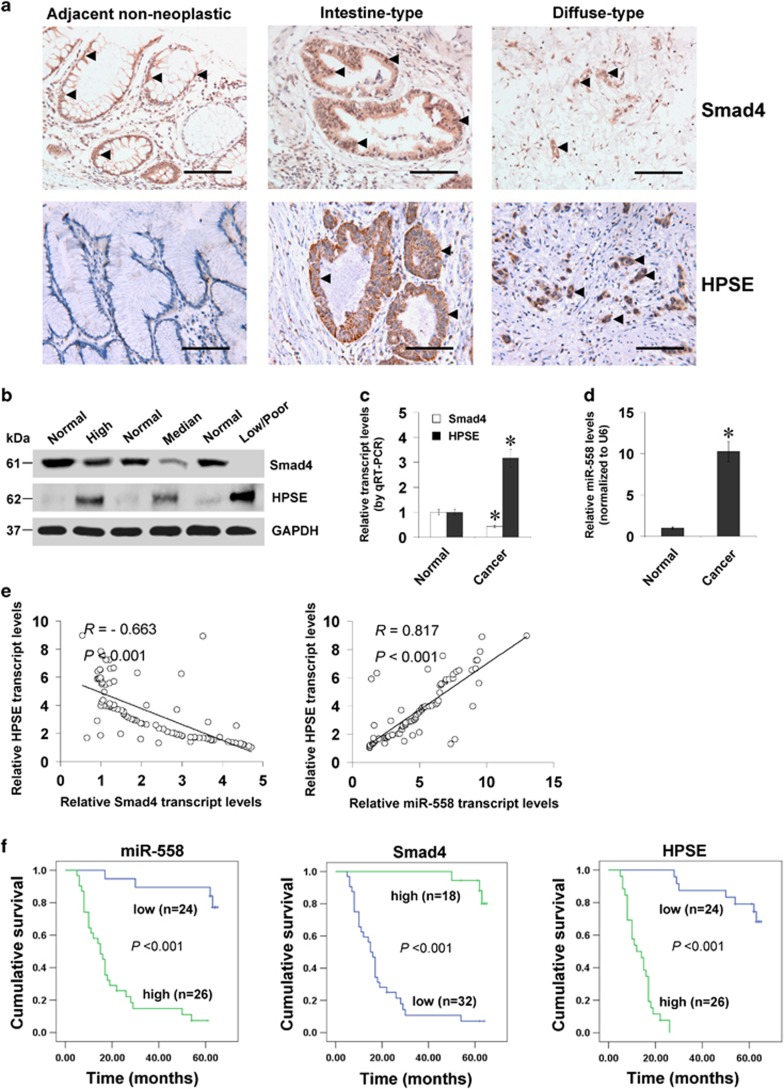
Smad4 and miR-558 are inversely or positively correlated with HPSE expression in gastric cancer tissues. (**a**) Immunohistochemical staining showing the Smad4 and HPSE expression in the tumour cells of gastric cancer tissues (arrowheads, brown). Scale bars: 100 *μ*m. (**b**) Western blot assay indicating the protein levels of Smad4 and HPSE in gastric cancer tissues with different differentiation, and those in normal gastric mucosa. (**c**) and (**d**) Real-time quantitative RT-PCR showing the transcript levels of *Smad4*, *HPSE* and *miR-558* in normal gastric mucosa (*n*=90) and gastric cancer tissues (*n*=90). (**e**) The correlation between *HPSE* transcript levels and *Smad4* or *miR-558* expression in gastric cancer tissues (*n*=90). (**f**) Kaplan–Meier survival curves of 50 well-defined gastric cancer cases with low or high expression of miR-558, Smad4 or HPSE. *P<0.01 versus normal

Mining the publicly available data derived from cBioPortal for Cancer Genomics (http://cbioportal.org) indicated low Smad4 mutation frequency in gastric cancer (Supplementary Figure S6A). To observe the expression of Smad4 in gastric cancer specimens, immunohistochemical staining was undertaken on paraffin-embedded sections from 50 well-established primary cases. The results indicated cytoplasmic and nuclear Smad4 expression in cancer cells (Figure 6a), which was detected in 22/50 (44.0%) cases, with weak staining in 4, moderate in 14, and intense in 4 (Supplementary Table S1). Lower Smad4 expression was observed in gastric cancer tissues with deeper gastric wall invasion (*P*<0.001), lymph node metastasis (*P*<0.001), distant metastasis (*P*=0.029) or advanced tumour-node-metastasis stage (*P*<0.001) (Supplementary Table S1). A negative correlation between Smad4 and HPSE immunoreactivity was noted in gastric cancer cases (correlation coefficient *R*=−0.614, *P*<0.001, Figure 6a and Supplementary Table S2). In 90 fresh gastric cancer specimens, lower Smad4 levels or higher HPSE expression were observed than those in normal gastric mucosa (Figures 6b and c), similar to results from Gene Expression Omnibus datasets (Supplementary Figures S6B and S6C). In contrast, miR-558 was up-regulated in gastric cancer tissues, when comparing with that in normal gastric mucosa (Figure 6d and Supplementary Figure S6D). Additionally, the BIRC6 levels were increased in gastric cancer specimens derived from public datasets (Supplementary Figure S6D). Notably, the expression levels of *Smad4* or miR-558 were inversely (*R*=−0.663, *P*<0.001, Figure 6e) and positively (*R*=0.817, *P*<0.001, Figure 6e) correlated with those of *HPSE* in gastric cancer tissues, respectively. Kaplan–Meier survival curves revealed that patients with high miR-558 levels (*P*<0.001), low Smad4 expression (*P*<0.001) or high HPSE levels (*P*<0.001) had lower survival probability, respectively (Figure 6f). These results indicated the under-expression of Smad4 and over-expression of miR-558 in gastric cancer tissues, which were inversely and positively correlated with the HPSE levels, respectively.

## Discussion

Smad4, first identified as a tumour suppressor in pancreatic cancer, is a key transcription factor of Smad family.^23^ Subsequent studies show that Smad4 is involved in the inhibitory functions of TGF-*β* signalling during the tumour progression.^24, 25^ *Smad4* deletion initiates the spontaneous tumorigenesis and promotes the Kras-initiated growth of lung cancers.^26^ *Smad4* deficiency mice are prone to develop polyps in the gastrointestinal tract, implicating its tumour suppressive functions.^27^ Inactivating mutation of *Smad4* gene is frequently identified in pancreatic cancer,^23^ cholangiocarcinoma^28^ and prostate cancer,^29^ and is associated with advanced stages and poor outcome of patients.^28, 29^ However, *Smad4* mutation is less frequently associated with breast cancer,^30^ esophageal cancer^31^ and gastric cancer.^31^ In this study, we searched the publicly available database cBioPortal, and found low frequency of *Smad4* mutation in gastric cancer. In addition, low Smad4 expression was associated with invasion, metastasis and tumour-node-metastasis stages in our series of gastric cancer patients. We demonstrated that Smad4 inhibited the growth, invasion, metastasis and angiogenesis of gastric cancer cells, and patients with low expression of Smad4 have lower survival probability, indicating the tumour suppressive functions of Smad4 during the progression of gastric cancer.

Human Smad4 protein, consisting of 552 amino acids, is able to transmit the TGF-*β* signalling,^24, 25^ and recognize the Smad-binding elements for transcriptional regulation of target genes.^32^ So far, many Smad4-regulated genes have been identified, including plasminogen activator inhibitor-1,^33^ *p21*^*Waf1*^,^34^ collagen type I alpha 2^35^ and platelet-derived growth factor B-chain.^36^ Previous studies have indicated that Smad4 suppresses the proliferation of pancreatic cancer initiating cells through transcriptional repression of aldehyde dehydrogenase 1A1.^37^ Ectopic expression of Smad4 induces the p21^waf1^ expression in breast cancer cells.^34^ In the current study, we found the inverse correlation between Smad4 and HPSE levels in gastric cancer specimens and cell lines. Importantly, we demonstrated that Smad4 directly bound to the target site within *HPSE* promoter to repress its expression, indicating the crucial functions of Smad4 in repressing the transcription of *HPSE*.

In this study, we noted the adjacent binding sites of miR-558 and Smad4 within the *HPSE* promoter. As a class of small non-coding RNAs, miRNAs mainly target the complementary sites within the 3'-untranslated regions and interact with AGO protein family to suppress translation or degrade mRNA.^38^ Recent studies indicate that endogenous miRNAs can recognize complementary genomic sites, and participate in the heterochromatin formation and transcriptional activation.^39, 40, 41^ For example, in prostate cancer cells, miR-373 activates the expression of E-cadherin through recognizing the target site within its promoter,^39^ while miR-205 targets the promoter of interleukin-24 and interleukin-32 to increase their expression.^40^ In addition, miR-744 recruits the RNA polymerase II and H3K4me3 on the *cyclin B1* promoter to activate its transcription in an AGO1-dependent manner.^41^ Our previous evidence has shown that miR-558 facilitates the transcription of *HPSE* through directly binding to its promoter in neuroblastoma.^22^ However, the roles of miR-558 in gastric cancer still remain to be elucidated. In this study, we found the up-regulation of miR-558 in gastric cancer tissues and cells, while its expression profile in gastric cell lines (HGE-17 and HGE-20) with true epithelial characteristics^42^ warrants further investigation. In addition, we demonstrated that miR-558 promoted the HPSE expression via attenuating the binding and repressive effects of Smad4 on *HPSE* promoter in gastric cancer cells. The findings that ectopic expression of miR-558 was able to rescue the gastric cancer cell lines from Smad4-inhibited biological behaviours indicate that the oncogenic functions of miR-558 are exerted, at least in part, through repressing the Smad4 activity in gastric cancer.

Since AGO1 is important for active chromatin remodelling at gene promoters induced by miRNA,^41^ we further observed the functions of AGO1 in miR-558-activated expression of HPSE in gastric cancer. Our evidence indicated that AGO1 was enriched surrounding the binding site of miR-558 within *HPSE* promoter in gastric cancer cells. In addition, treatment of gastric cancer cells with RNase H (degrading the RNA within RNA-DNA hybrid)^43^ inhibited the enrichment of AGO1 induced by miR-558, indicating the miR-558-mediated recruitment of AGO1 on *HPSE* promoter. However, over-expression of miR-558 or knockdown of AGO1 did not affect the enrichment of epigenetic markers. Instead, the miR-558-attenuated Smad4 binding to *HPSE* promoter was abolished by knockdown of *AGO1*. We suspect that miR-558 may form complex with AGO1 to bring in steric hindrance effects to repress the binding of Smad4, which warrants further investigation.

In conclusion, we have shown that Smad4 is under-expressed and suppresses the transcription of *HPSE* through directly binding to its promoter in gastric cancer. Furthermore, miR-558 is up-regulated in gastric cancer, and promotes the transcription of *HPSE* via abolishing the binding of Smad4 to its promoter, resulting in increased *in vitro* and *in vivo* growth, invasion, metastasis and angiogenesis of gastric cancer cells. Our findings reveal the mechanisms of *HPSE* gene expression associated with gastric cancer progression, and suggest that miR-558 and Smad4 are potential therapeutic targets of gastric cancer.

## Materials and Methods

### Cell culture

Human gastric cancer cell lines AGS (CRL-1739), SGC-7901, MKN-28 and MKN-45, human primary stomach epithelial HPSEC cells (H-6039), SV40-immortalized normal gastric epithelial GES-1 cells and human endothelial cell line HUVEC (CRL-1730) were purchased from the American Type Culture Collection (Rockville, MD, USA), Cell Biologics Inc. (Chicago, IL, USA) and Type Culture Collection of Chinese Academy of Sciences (Shanghai, China), authenticated by the supplier, and used within 6 months after resuscitation of frozen aliquots. Cell lines were grown at 37 °C in a humidified atmosphere of 5% CO_2_, with RPMI1640 medium (Life Technologies, Inc., Gaithersburg, MD, USA) or M6621 medium (Cell Biologics Inc.) containing 10% fetal bovine serum (Life Technologies, Inc.), penicillin (100 U/ml) and streptomycin (100 *μ*g/ml).

### Gene over-expression and knockdown

Human *Smad4* expression construct was a gift from Dr Anna Coppa.^44^ Human *HPSE* cDNA (1632 bp) was amplified from NB tissue (Supplementary Table S3), and inserted into pcDNA3.1 (Invitrogen, Carlsbad, CA, USA). The oligonucleotides encoding shRNAs against *Smad4* were inserted into GV102 (Genechem Co., Ltd, Shanghai, China; Supplementary Table S3). The 21-nucleotide siRNAs specific for *AGO1*, *AGO2*, *AGO3*, *AGO4* and *HPSE*^45, 46^ were chemically synthesized (RiboBio Co., Ltd, Guangzhou, China; Supplementary Table S3). Transfection of *Smad4* or *Smad4* shRNA vectors was performed using Lipofectamine 2000 (Invitrogen). After selecting for puromycin (Invitrogen) resistance, stable cell lines were obtained.

### Western blot

Protein from cell lines and tissues were extracted using 1 × cell lysis buffer (Promega, Madison, WI, USA). The SDS-PAGE electrophoresis and immunoblotting were performed as previously reported,^22, 47, 48, 49, 50, 51^ with antibodies specific for Smad4, HPSE, VEGF, glyceraldehyde-3-phosphate dehydrogenase (GAPDH; Santa Cruz Biotechnology, Santa Cruz, CA, USA), AGO1, AGO2, AGO3 and AGO4 (Cell Signaling Technology, Inc., Danvers, MA, USA).

### Real-time quantitative RT-PCR

Isolation of total RNA from cell lines and tissues was performed using RNeasy Mini Kit (Qiagen Inc., Valencia, CA, USA). After the reverse transcription reactions with Transcriptor First Strand cDNA Synthesis Kit (Roche, Indianapolis, IN, USA), real-time PCR was conducted using primers (Supplementary Table S4) and SYBR Green PCR Master Mix (Applied Biosystems, Foster City, CA, USA). The transcript levels of genes were analysed by 2^−△△Ct^ method.

### Prediction and measurement of miRNA

The algorithm microPIR^20^ was applied to analyse the potential miRNA targeting sites within *HPSE* promoter. The miRNA-specific stem-loop primer, PCR primers (Supplementary Table S4), and Bulge-Loop^TM^ miRNAs qPCR Primer Set (RiboBio Co. Ltd) were used to synthesize the cDNA and measure the levels of mature miR-558. The results were analysed by normalizing the miRNA levels to those of U6 snRNA.

### Over-expression and knockdown of miRNA

Based on the sequence in the miRNA Registry database,^52^ the construct of miR-558 precursor was established by inserting the encoding oligonucleotides (Supplementary Table S3) into pcDNA3.1(-) (Invitrogen). After selecting for neomycin (Invitrogen) resistance, the miR-558 over-expressing stable cancer cell lines were obtained. To knockdown of miR-558, confluent cells were transfected with negative control or anti-miR-558 inhibitors (RiboBio Co. Ltd) using Lipofectamine 2000 (Invitrogen).

### Promoter activity assay

The luciferase reporter of human *HPSE* promoter was a kind gift from Dr Xiulong Xu (Rush University Medical Center).^10^ Human *VEGF* promoter (−2000/+168 bp) luciferase reporter was obtained from Genechem Co., Ltd. The GeneTailor^TM^ Site-Directed Mutagenesis System (Invitrogen) and PCR primers (Supplementary Table S3) were applied to generate the constructs with mutant binding sites of Smad4 or miR-558. The activity of *HPSE* promoter was measured by dual-luciferase assay.^22, 45, 50, 51^

### Nascent transcription detection

The nascent transcription of genes within cancer cells were measured by nuclear run-on assay.^22, 45^ After incorporation of biotin-16-uridine-5′-triphosphate, the Trizol and agarose-conjugated streptavidin beads (Invitrogen) were applied for extraction of total and biotinylated nascent RNA. Real-time RT-PCR was performed as above described.

### ChIP assay

The EZ-ChIP kit (Upstate Biotechnology, Temacula, CA, USA) was applied in ChIP assay,^45, 50, 51, 53^ with antibodies specific for Smad4, AGO1, H3K9me2, H3K27me3, or H3K4me3 (Upstate Biotechnology). Prior to immunoprecipitation, the RNase H (10 U) or RNase A (20 μg) was used to treat the lysates. The SYBR Green PCR Master Mix and primer sets (Supplementary Table S4) were applied for real-time qPCR.

### *In vitro* cell viability, growth, invasion and angiogenesis assays

The 2-(4,5-dimethyltriazol-2-yl)-2,5-diphenyl tetrazolium bromide (MTT; Sigma, St. Louis, MO, USA) colorimetric,^45^ soft agar,^22, 51^ matrigel invasion,^47, 48, 50, 51, 53, 54^ and tube formation^22, 47^ assays were performed to measure the *in vitro* viability, growth, invasion and angiogenesis capabilities of cancer cells.

### *In vivo* growth and metastasis assay

The Animal Care Committee of Tongji Medical College approved all the experiments in BALB/c nude mice (approval number: Y20080290). The 2-month-old male BALB/c nude mice were blindly randomized into groups (*n*=5 for each group) and applied in the *in vivo* tumour growth and experimental metastasis studies.^47, 48, 50^

### Clinical specimens and measurement

The Institutional Review Board of Tongji Medical College approved the study (approval number: 2011-S085). The fresh tumour and adjacent normal gastric specimens from 90 well-established primary gastric cancer cases were collected at surgery, validated by pathological diagnosis, stored at −80 °C, and used for detection of gene expression by western blot and real-time RT-PCR. The demographic and clinicopathological details of subtotal 50 patients were indicated in Supplementary Table S1.

### Immunohistochemical staining

Immunohistochemical staining was undertaken as described previously,^22, 50, 51, 55^ with antibodies specific for Smad4, HPSE and CD31 (Santa Cruz Biotechnology; 1:200 dilutions).

### Statistical analysis

All data were presented as mean±standard error of the mean (S.E.M.). To compare the gene expression and analyse the relationship among gene expression, the χ^2^ analysis, Fisher exact probability analysis and Pearson's coefficient correlation assay were applied. The Kaplan–Meier method and log-rank test were applied to assess survival rates and difference. The *t* test or analysis of variance (ANOVA) was used to determine the difference of tumour cells.

## Abbreviations

AGO1: Argonaute 1
AGO2: Argonaute 2
AGO3: Argonaute 3
AGO4: Argonaute 4
ANOVA: analysis of variance
anti-NC: negative control inhibitor
BIRC6: baculoviral IAP repeat containing 6
ChIP: chromatin immunoprecipitation
ChIP-seq: chromatin immunoprecipitation sequencing
GAPDH: glyceraldehyde-3-phosphate dehydrogenase
H3K27me3: histone H3 lysine 27 trimethylation
H3K4me3: histone H3 lysine 4 trimethylation
H3K9me2: histone H3 lysine 9 dimethylation
HPSE: heparanase
miR-558: microRNA-558
Mock: empty vector
MTT: 2-(4,5-dimethyltriazol-2-yl)-2,5-diphenyl tetrazolium bromide
Mut: mutant
qPCR: quantitative PCR
SEM: standard error of the mean
shRNA: short hairpin RNA
sh-Scb: scramble short hairpin RNA
sh-Smad4: short hairpin RNA targeting Smad4
si-AGO1: AGO1-specific small interfering RNA
siRNAs: small interfering RNAs
si-Scb: scramble small interfering RNA
Smad4: SMAD family member 4
TSS: transcription start site
VEGF: vascular endothelial growth factor
WT: wild type
