# Supplementary material for: miRNA-558 promotes gastric cancer progression through attenuating Smad4-mediated repression of heparanase expression
Source: Cell Death Dis. 2016 Sep 29;7(9):e2382–. doi: 10.1038/cddis.2016.293 (PMC5059886; doi:10.1038/cddis.2016.293)
Supplement: Supplementary Table S1 [file cddis2016293x1.doc]

**Supplementary Table S1 Smad4 and HPSE expression in human gastric cancer tissues**

| **Clinicopathologic factor** | **Total** |  | **Smad4 expression** | |  | **HPSE expression** | |
| --- | --- | --- | --- | --- | --- | --- | --- |
|  | **n (%)** |  | **n (%)** | ***P*-value** |  | **n (%)** | ***P*-value** |
| **Age (years)** |  |  |  |  |  |  |  |
| ≤60 | 26 (52.0) |  | 11 (42.3) | 0.802 |  | 20 (76.9) | 0.874 |
| >60 | 24 (48.0) |  | 11 (45.8) |  |  | 18 (75.0) |  |
| **Sex** |  |  |  |  |  |  |  |
| Male | 35 (70.0) |  | 15 (42.9) | 0.804 |  | 26 (74.3) | 0.665 |
| Female | 15 (30.0) |  | 7 (46.7) |  |  | 12 (80.0) |  |
| **Size (diameter)** |  |  |  |  |  |  |  |
| ≤6 cm | 33 (66.0) |  | 14 (42.4) | 0.754 |  | 25 (75.8) | 0.955 |
| >6 cm | 17 (34.0) |  | 8 (47.1) |  |  | 13 (76.5) |  |
| **Laurén classification** |  |  |  |  |  |  |  |
| Intestinal type | 29 (58.0) |  | 13 (44.8) | 0.890 |  | 22 (75.8) | 0.979 |
| Diffuse type | 21 (42.0) |  | 9 (42.9) |  |  | 16 (76.2) |  |
| **Gastric wall invasion** |  |  |  |  |  |  |  |
| T1/T2 | 18 (36.0) |  | 15 (83.3) | <0.001 |  | 8 (44.4) | <0.001 |
| T3/T4 | 32 (64.0) |  | 7 (21.9) |  |  | 30 (93.8) |  |
| **Lymph node metastasis** |  |  |  |  |  |  |  |
| Negative | 18 (36.0) |  | 16 (88.9) | <0.001 |  | 9 (50.0) | 0.001 |
| Positive | 32 (64.0) |  | 6 (18.8) |  |  | 29 (90.6) |  |
| **Distant metastasis** |  |  |  |  |  |  |  |
| Negative | 38 (76.0) |  | 20 (52.6) | 0.029 |  | 26 (68.4) | 0.026 |
| Positive | 12 (24.0) |  | 2 (16.7) |  |  | 12 (100.0) |  |
| **TNM stage** |  |  |  |  |  |  |  |
| I/II | 17 (34.0) |  | 17 (100.0) | <0.001 |  | 8 (47.1) | 0.001 |
| III/IV | 33 (66.0) |  | 5 (15.2) |  |  | 30 (90.9) |  |

Smad4, SMAD family member 4; HPSE, heparanase; TNM, tumor-node-metastasis.
