# Supplementary material for: miRNA-558 promotes gastric cancer progression through attenuating Smad4-mediated repression of heparanase expression
Source: Cell Death Dis. 2016 Sep 29;7(9):e2382–. doi: 10.1038/cddis.2016.293 (PMC5059886; doi:10.1038/cddis.2016.293)
Supplement: Supplementary Table S2 [file cddis2016293x2.doc]

**Supplementary Table S2 Correlation between the expression of Smad4 and HPSE**

|  |  | **Smad4 expression** | | |  |  |  |  |
| --- | --- | --- | --- | --- | --- | --- | --- | --- |
|  |  | Low |  | High |  | *R*-value |  | *P*-value |
| **HPSE expression** | |  |  |  |  |  |  |  |
| Low | | 8 |  | 16 |  | - 0.614 |  | <0.001 |
|  | |  |  |  |  |  |  |  |
| High | | 24 |  | 2 |  |  |  |  |

Smad4, SMAD family member 4; HPSE, heparanase; Pearson’s correlation coefficient was applied to determine the expression correlation.
