# Supplementary material for: miRNA-558 promotes gastric cancer progression through attenuating Smad4-mediated repression of heparanase expression
Source: Cell Death Dis. 2016 Sep 29;7(9):e2382–. doi: 10.1038/cddis.2016.293 (PMC5059886; doi:10.1038/cddis.2016.293)
Supplement: Supplementary Table S3 [file cddis2016293x3.doc]

**Supplementary Table S3 Oligonucleotide sets used for constructs, inhibitors, short hairpin RNAs,**

**and small interfering RNAs**

| **Oligo Set** | **Sequences** |
| --- | --- |
| pre-miR-558 | 5'-TGCTGTGAGCTGCTGTACCAAAATGTTTTGGCCACTGACTGACATTTTGGTACAGCAGC  TCA-3' (sense); |
|  | 5'-CCTGTGAGCTGCTGTACCAAAATGTCAGTCAGTGGCCAAAACATTTTGGTACAGCAGCT  CAC-3' (antisense) |
| pre-miR-NC | 5'-TGCTGAAATGTACTGCGCGTGGAGACGTTTTGGCCACTGACTGACGTCTCCACGCAGTA  CATTT-3' (sense); |
|  | 5'-CCTGAAATGTACTGCGTGGAGACGTCAGTCAGTGGCCAAAACGTCTCCACGCGCAGTAC  ATTTC-3' (antisense) |
| pcDNA3.1-Smad4 | 5'-CGCGGATCCATGGACAATATGTCTATTACG-3' (sense); |
|  | 5'-CCGCTCGAGTCAGTCTAAAGGTTGTGGGTC-3' (antisense) |
| pcDNA3.1-HPSE | 5'-CGGGGTACCATGCTGCTGCGCTCGAAGCCTG-3' (sense); |
|  | 5'-CCGCTCGAGTCAGATGCAAGCAGCAACTTTGG-3' (antisense) |
| pGL3-HPSE mut  (ΔmiR-558) | 5'-TTGGTATGACTGGGCATGGCCTCTTGCGCTCTGCCTGCAAT-3' (sense); |
|  | 5'-GCCATGCCCAGTCATACCAATTTCTTTAAC-3' (antisense) |
| pGL3-HPSE mut  (ΔSmad4) | 5'-TGTGAGGCCGCTGAGGGCAGATCGCGAGGTCAGGAGATTGAGACC-3' (sense); |
|  | 5'-GCGATCTGCCCTCAGCGGCCTCACAAAATGCTAGGATTGCAGGCA-3' (antisense) |
| Anti-NC | RiboBio |
| Anti-miR-558 | RiboBio |
| sh-Scb | 5'-CCGGGCGAACGATCGAGTAAACGGACTCGAGTCCGTTTACTCGATCGTTCGCTTTTT-3'  (sense); |
|  | 5'-AATTCAAAAAGCGAACGATCGAGTAAACGGACTCGAGTCCGTTTACTCGATCGTTCGC-3'  (antisense) |
| sh-Smad4-1 | 5'-CCGGGCAGACAGAAACTGGATTAAACTCGAGTTTAATCCAGTTTCTGTCTGCTTTTT-3'  (sense); |
|  | 5'-AATTCAAAAAGCAGACAGAAACTGGATTAAACTCGAGTTTAATCCAGTTTCTGTCTGC-3'  (antisense) |
| sh-Smad4-2 | 5'-CCGGCCTGAGTATTGGTGTTCCATTCTCGAGAATGGAACACCAATACTCAGGTTTTT-3'  (sense); |
|  | 5'-AATTCAAAAACCTGAGTATTGGTGTTCCATTCTCGAGAATGGAACACCAATACTCAGG-3'  (antisense) |
| si-Scb | 5'-GAACGAUCGAGUAAACGGAtt-3' (sense); |
|  | 5'-UCCGUUUACUCGAUCGUUCtt-3' (antisense) |
| si-AGO1 | 5'-GAGAAGAGGUGCUCAAGAAtt-3' (sense); |
|  | 5'-UUCUUGAGCACCUCUUCUCtt-3' (antisense) |
| si-AGO2 | 5'-GCACGGAAGUCCAUCUGAAtt-3' (sense); |
|  | 5'-UUCAGAUGGACUUCCGUGCtt-3' (antisense) |
| si-AGO3 | 5'-GGAACUUCUUAUUCAAUUUtt-3' (sense); |
|  | 5'-AAAUUGAAUAAGAAGUUCCtt-3' (antisense) |
| si-AGO4 | 5'-CUACAGCUAAUAGUGGUUAtt-3' (sense); |
|  | 5'-UAACCACUAUUAGCUGUAGtt-3' (antisense) |
| si-HPSE | 5'-CTCTUUUGUTGGTGGUTGUtt-3' (sense); |
|  | 5'-CTCTUUUGUTGGTGGUTGUtt-3' (antisense) |

Pre-miR-NC, negative control pre-miRNA; Smad4, SMAD family member 4; HPSE, heparanase; Anti-NC, negative control inhibitor; sh-Scb, scramble short hairpin RNA; si-Scb, scramble small interfering RNA; AGO1, argonaute 1; AGO2, argonaute 2; AGO3, argonaute 3; AGO4, argonaute 4.
