# Supplementary material for: miRNA-558 promotes gastric cancer progression through attenuating Smad4-mediated repression of heparanase expression
Source: Cell Death Dis. 2016 Sep 29;7(9):e2382–. doi: 10.1038/cddis.2016.293 (PMC5059886; doi:10.1038/cddis.2016.293)
Supplement: Supplementary Table S4 [file cddis2016293x4.doc]

**Supplementary Table S4 Primer sets used for qRT-PCR, nuclear run-on, and ChIP**

| **Primer set** | **Primers** | **Sequence** | **Product size (bp)** | **Application** |
| --- | --- | --- | --- | --- |
| HPSE | Forward | 5'-GAATGGACGGACTGCTAC-3' | 261 | qRT-PCR |
|  | Reverse | 5'-CCAAAGAATACTTGCCTCA-3' |  | nuclear run-on |
| VEGF | Forward | 5'-ATGACGAGGGCCTGGAGTGT-3' | 226 | qRT-PCR |
|  | Reverse | 5'-CATTTACACGTCTGCGGATCT-3' |  |  |
| Smad4 | Forward | 5'-TGCCTCACCACCAAAACGG-3' | 255 | qRT-PCR |
|  | Reverse | 5'-CCAAACAAAAGCGATCTCCTCC-3' |  |  |
| GAPDH | Forward | 5'-AGAAGGCTGGGGCTCATTTG-3' | 258 | qRT-PCR |
|  | Reverse | 5'-AGGGGCCATCCACAGTCTTC-3' |  |  |
| miR-558 | Forward | RiboBio |  | qRT-PCR |
|  | Reverse | RiboBio |  |  |
| U6 | Forward | RiboBio |  | qRT-PCR |
|  | Reverse | RiboBio |  |  |
| HPSE ChIP | Forward | 5'-AAATTGGTATGACTGGGCATGG-3' | 200 | ChIP |
| (-2347~-2148) | Reverse | 5'-TCAGCCTCCCGAGTAGCTGGGACTA-3' |  |  |

HPSE, heparanase; VEGF, vascular endothelial growth factor; Smad4, SMAD family member 4; GAPDH, glyceraldehyde 3-phosphate dehydrogenase.
