# Supplementary figures and images for: miRNA-558 promotes gastric cancer progression through attenuating Smad4-mediated repression of heparanase expression
Source: Cell Death Dis. 2016 Sep 29;7(9):e2382–. doi: 10.1038/cddis.2016.293 (PMC5059886; doi:10.1038/cddis.2016.293)

# Supplementary Figure S1

A

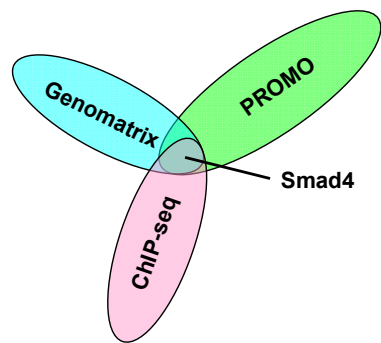

B

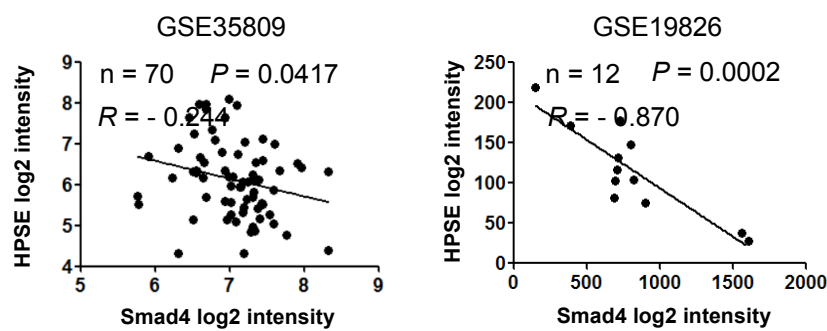

C

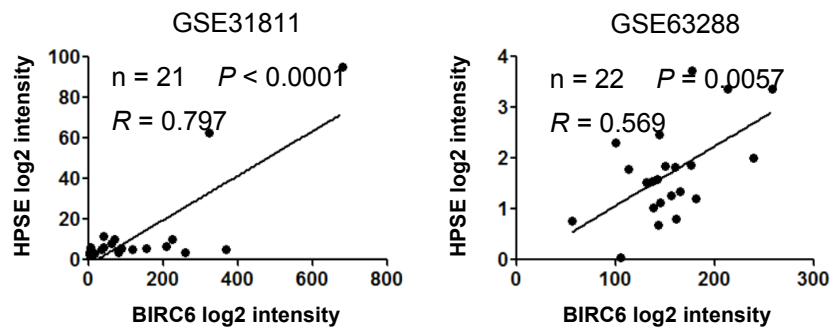

Supplement: Supplementary Figure S1 [file cddis2016293x5.pdf]

# Supplementary Figure S2

A

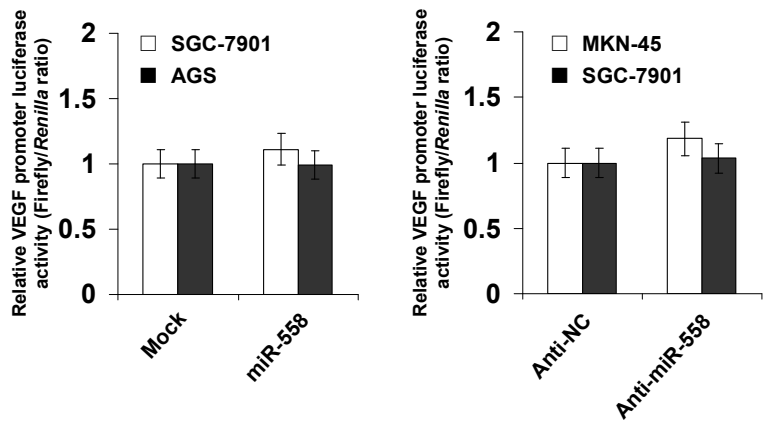

B

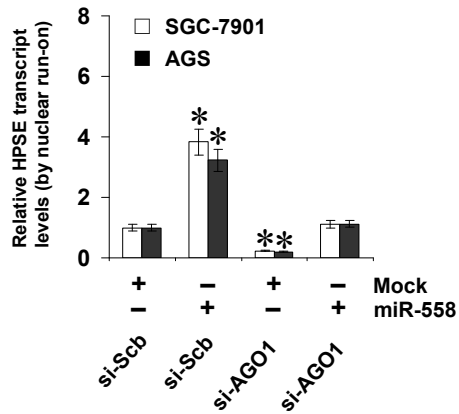

Supplement: Supplementary Figure S2 [file cddis2016293x6.pdf]

# Supplementary Figure S3

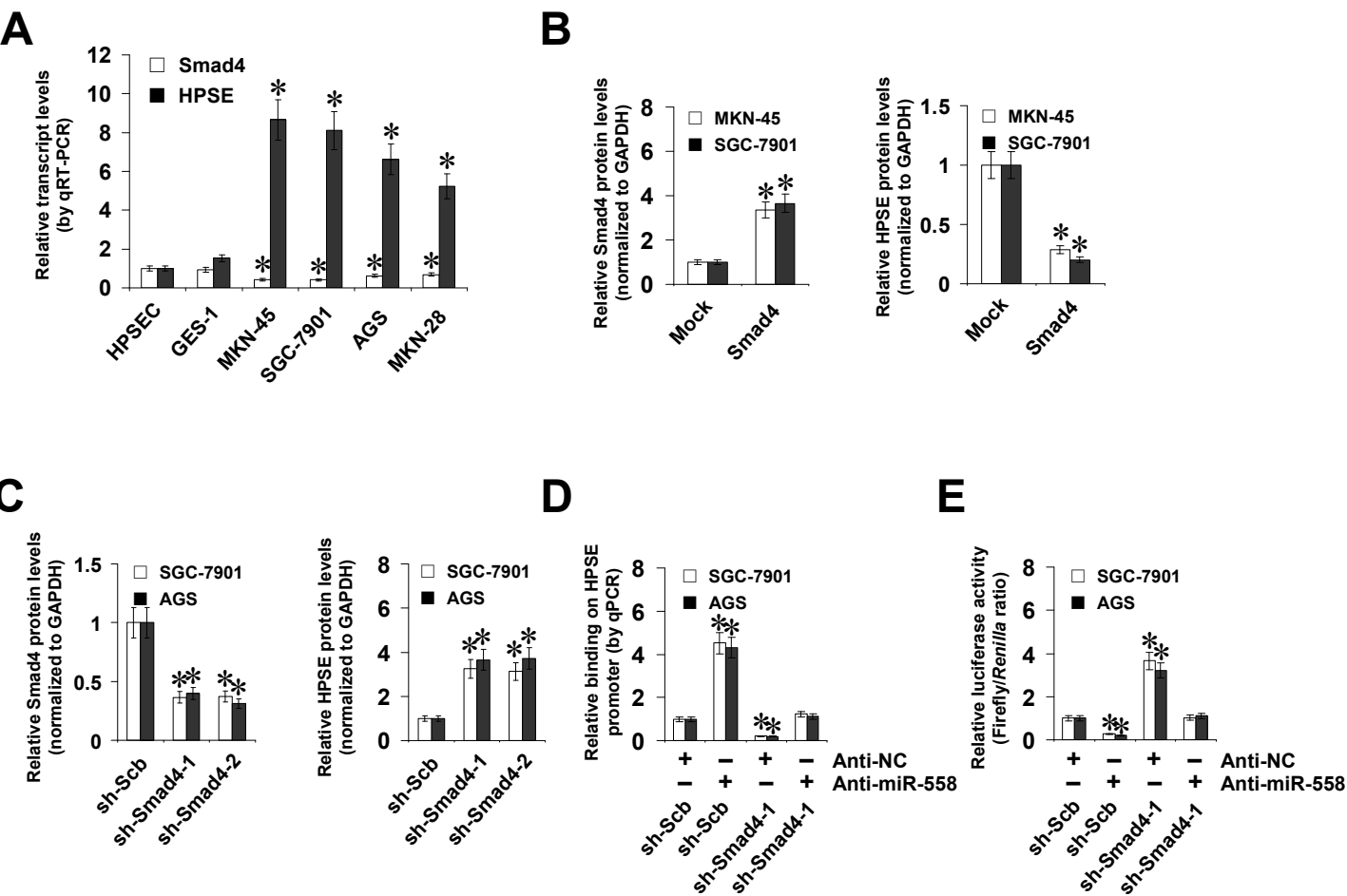

Supplement: Supplementary Figure S3 [file cddis2016293x7.pdf]

# Supplementary Figure S4

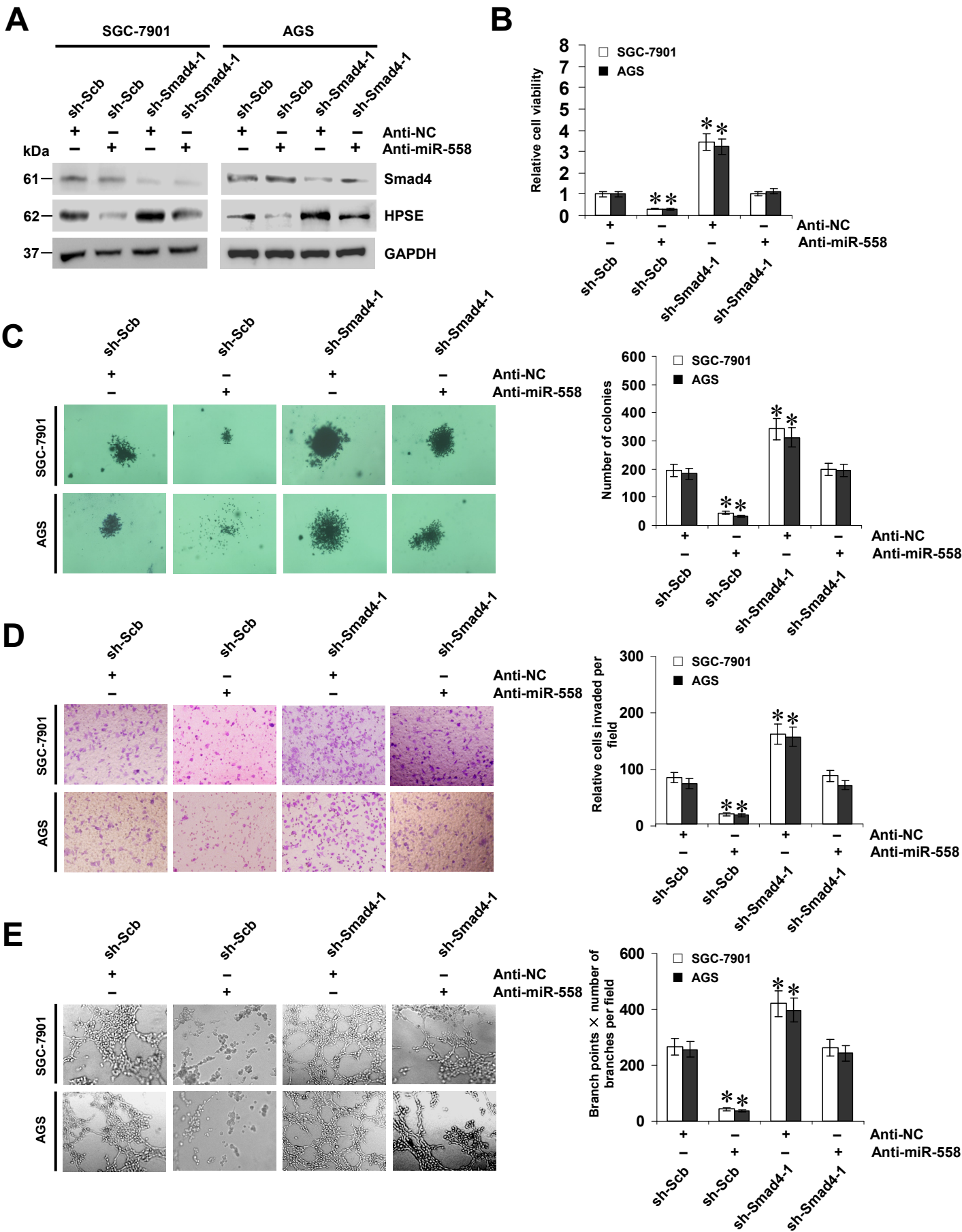

Supplement: Supplementary Figure S4 [file cddis2016293x8.pdf]

Supplementary Figure S5

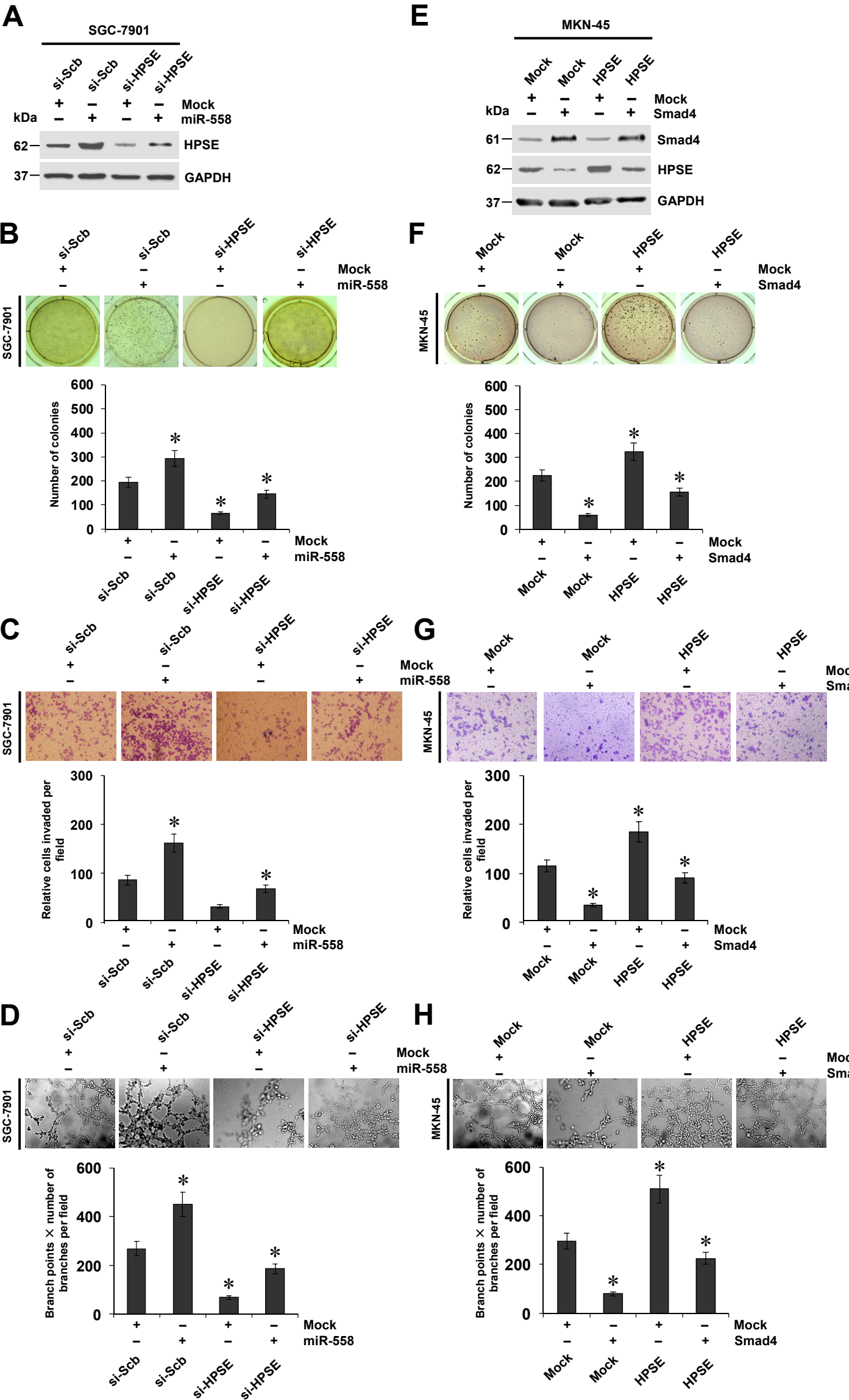

Supplement: Supplementary Figure S5 [file cddis2016293x9.pdf]

# Supplementary Figure S6

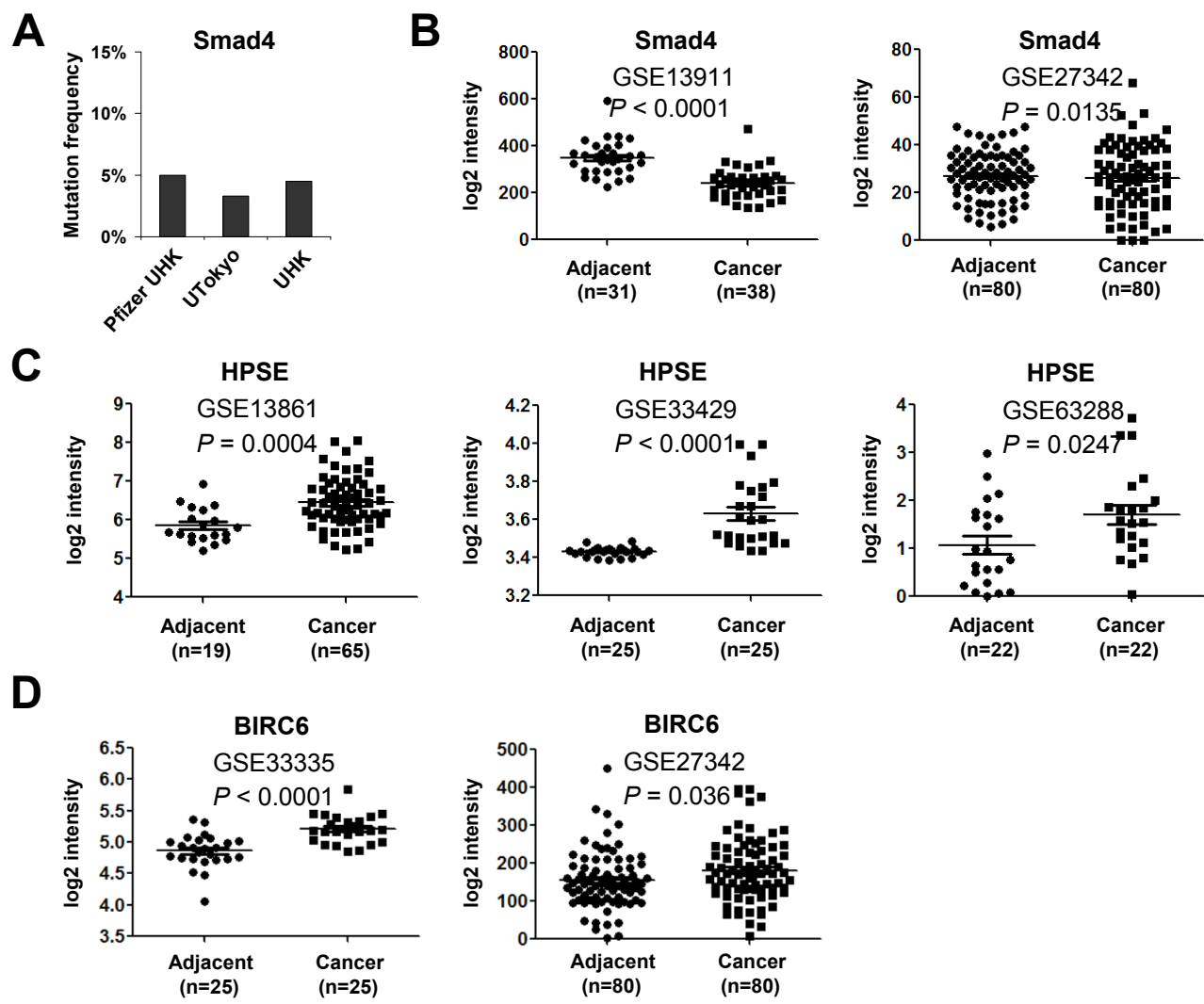

Supplement: Supplementary Figure S6 [file cddis2016293x10.pdf]
