# Supplementary material for: miRNA-558 promotes gastric cancer progression through attenuating Smad4-mediated repression of heparanase expression
Source: Cell Death Dis. 2016 Sep 29;7(9):e2382–. doi: 10.1038/cddis.2016.293 (PMC5059886; doi:10.1038/cddis.2016.293)
Supplement: Supplementary Figure Legends [file cddis2016293x11.doc]

**Supplementary Figure Legends**

**Supplementary Figure S1 Mining publicly available datasets. A**, over-lapping analysis of computational programs (Genomatrix and PROMO) and ChIP-seq dataset (GSE27526) revealing Smad4 as a transcription factor binding to *HPSE* promoter region (chr4:84255936-84259422). **B** and **C**, the correlation between HPSE and Smad4 or BIRC6 levels in gastric cancer tissues derived from public GEO datasets.

**Supplementary Figure S2 Effects of miR-558 on *VEGF* promoter activity and HPSE expression in gastric cancer cells. A**, dual-luciferase assay indicating the *VEGF* promoter activity in cancer cells transfected as indicated. **B**, nuclear run-on assay showing the nascent *HPSE* transcript levels in gastric cancer cells transfected as indicated. * *P*<0.01 vs. mock+si-Scb.

**Supplementary Figure S3 HPSE and Smad4 expression in gastric cancer cells.** Real-time quantitative RT-PCR (**A**), quantification of western blot (**B** and **C**), ChIP (**D**), and dual-luciferase (**E**) assays indicating the HSPE and Smad4 expression, Smad4 binding, and *HPSE* promoter activity in cancer cells transfected as indicated. **P*<0.01 vs. HPSEC, mock, sh-Scb, or sh-Scb+anti-NC.

**Supplementary Figure S4 Effects of miR-558 and Smad4 on gastric cancer cells.** Western blot (**A**), MTT colorimetric (**B)**, soft agar (**C**), matrigel invasion (**D**), and tube formation (**E**) assays indicating gene expression, viability, growth, invasion, and angiogenesis of cancer cells transfected with sh-Scb, sh-Smad4, anti-NC, or anti-miR-558 inhibitor (100 nmol/L). **P*<0.01 vs. sh-Scb+anti-NC.

**Supplementary Figure S5 miR-558 and Smad4 exert functions through regulating HPSE expression.** Western blot (**A** and **E**), soft agar (**B** and **F**), matrigel invasion (**C** and **G**), and tube formation (**D** and **H**) assays indicating gene expression, growth, invasion, and angiogenesis of cancer cells transfected as indicated. **P*<0.01 vs. mock+si-Scb or mock.

**Supplementary Figure S6 Gene expression profiles in public datasets. A**, the data derived from cBioPortal for Cancer Genomics (http://cbioportal.org) showing the Smad4 mutation frequency in gastric cancer cohorts. **B**, **C**, and **D**, the expression of Smad4, HPSE, and BIRC6 in gastric cancer and adjacent normal tissues derived from GEO datasets.
